# Synthesis, Characterization and Pharmacological Evaluation of 1-(2-Chloro-6-Fluorophenyl)-5-Methylindolin-2-One: A New Anti-Inflammatory Compound with Reduced Gastric Ulceration Properties 

**DOI:** 10.3390/molecules15118039

**Published:** 2010-11-08

**Authors:** Jean Leandro dos Santos, Rafael Chelucci, Richard Chiquetto, Man Chin Chung, Michel Leandro Campos, Rosangela Gonçalves Peccinini

**Affiliations:** 1 Lapdesf - Laboratório de Pesquisa e Desenvolvimento de Fármacos, Departamento de Fármacos e Medicamentos, Faculdade de Ciências Farmacêuticas – UNESP Rodovia Araraquara Jaú Km. 01, 14801-902, Araraquara, SP, Brazil; 2 Departamento de Princípios Ativos, Naturais e Toxicologia, Faculdade de Ciências Farmacêuticas - UNESP Rodovia Araraquara Jaú Km. 01, 14801-902, Araraquara, SP, Brazil

**Keywords:** pro-drug, lactam, lumiracoxib, anti-inflammatory

## Abstract

The new compound 1-(2-chloro-6-fluorophenyl)-5-methylindolin-2-one (**1**), designed using the prodrug approach, was easily obtained in 85% yield and characterized by nuclear magnetic resonance, elemental analysis, mass spectrometry and infrared spectroscopy. The lactam **1** showed anti-inflammatory and analgesic activity comparable to that of the COX-2 inhibitor lumiracoxib, without gastro-ulceration effects. Stability studies demonstrated that the lactam function was stable and did not hydrolyze in pH 1.2 or 7.4. Furthermore, using a thioglycollate-induced peritonitis model, compound **1** was shown to inhibit cell migration by 50.4%, while lumiracoxib inhibited it by 18%. This compound represents a new non-ulcerogenic prototype for the treatment of chronic inflammatory diseases.

## 1. Introduction

Nonsteroidal anti-inflammatory drugs (NSAIDs) are used for the treatment of inflammatory and rheumatic diseases. The common mechanism of NSAIDs involves the non-selective inhibition of cyclooxygenases (COX-1 and COX-2) preventing the biosynthesis of prostaglandins (PGs). The COX-1 enzyme is the constitutive isoform, and it provides the normal production of PGs. The enzyme has an important role in homeostasis and gastroprotection, and this enzyme inhibition, caused by the long-term use of drugs, is associated with gastrotoxicity. This side effect produced by NSAIDs involves two different mechanisms: inhibition of prostaglandin synthesis in the stomach and a local action exerted by direct contact of the drugs with the gastric mucosa [1].

**Scheme 1:**
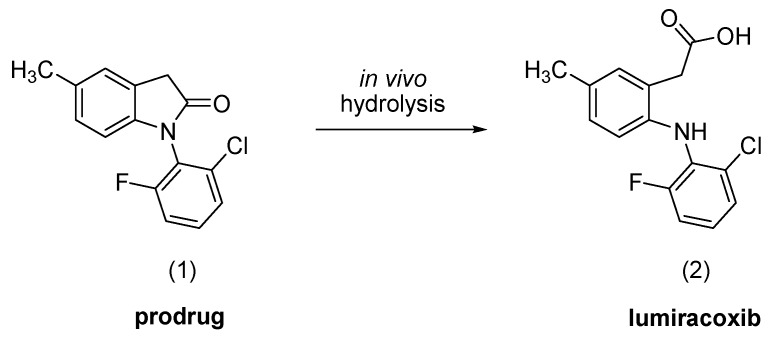
*In vivo* prodrug conversion to lumiracoxib.

As part of our ongoing program to identify new anti-inflammatory drug candidates with reduced gastric ulceration properties, we describe herein the synthesis and the preliminary biological evaluation of the compound 1-(2-chloro-6-fluorophenyl)-5-methylindolin-2-one (**1**), designed as a lumiracoxib prodrug. 

## 2. Results and Discussion

### 2.1. Synthesis

The title compound was prepared through an intramolecular reaction of lumiracoxib at room temperature for 1 h using 1-ethyl-3-[3-dimethylaminopropyl]carbodiimide hydrochloride (EDC) as coupling reagent. After, the reaction mixture was diluted with dichloromethane and washed with 0.2 M bicarbonate solution. The solvent was removed by evaporation, resulting in 85% yield of 1-(2-chloro-6-fluorophenyl)-5-methylindolin-2-one (**1**). The purity of the synthesized compound was checked by thin layer chromatography (TLC) and elemental analysis. The elemental analysis results were within ± 0.4% of the theoretical values. The structure was characterized by nuclear magnetic resonance (NMR), infrared spectroscopy (IR) and mass spectrometry (MS). The ^1^H-NMR spectrum showed the methyl group and the methylene protons at δ 2.35 (s, 3H) and 3.81 (s, 2H), respectively. The aromatic protons appeared at δ 6.83-7.4. The IR spectrum of compound **1 **showed the presence of a lactam carbonyl at 1.736 cm^−1^. The C-F and C-Cl stretching appeared at 1.150 cm^−1^ and 783 cm^−1^, respectively.

### 2.2. Pharmacological Evaluation

#### 2.2.1. Anti-inflammatory activity and ulcerogenicity studies

**Figure 1:**
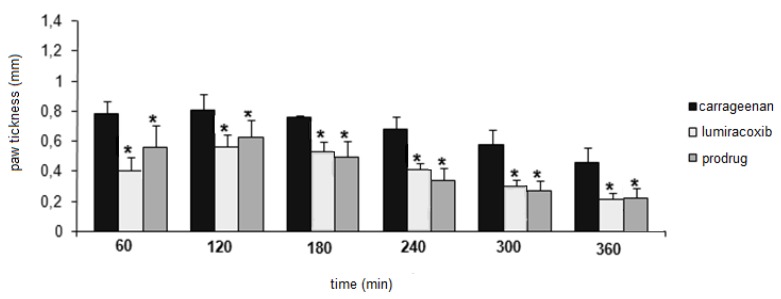
Anti-inflammatory activity in carrageenan-induced paw edema in rats (data are represented as mean ± S.E.M., *n* = 6, * *P* < 0.05 related to carrageenan group; dose of compounds = 300 μmol/kg).

Figure 1 demonstrates the inhibition of swelling rat paw edema induced by carrageenan after oral administration of the tested drugs. The *in vivo* result shows that 1-(2-chloro-6-fluorophenyl)-5-methylindolin-2-one (**1**) presented anti-inflammatory activity comparable with lumiracoxib, the parental drug used as control. From the first hour, the anti-inflammatory activity of lactam **1 **was statistically different when compared to the carrageenan group. All results were statistically different when compared to an aqueous solution of sodium carboxymethylcellulose (0.5% w/v), used as control (data not shown).

The ulcerogenicity study was carried out using rat gastric mucosa after a single oral administration of the compounds at 300 μmol/Kg^−1^ of each drug. The lesions were examined using a 4 × binocular magnifier and the results were obtained with an average of six animals analyzed per group. Table 1 demonstrates that the lactam 1-(2-chloro-6-fluorophenyl)-5-methylindolin-2-one (**1**) was unable to induce gastric ulceration. Diclofenac and lumiracoxib were used as COX-1 and COX-2 inhibitor controls, respectively. For each stomach, the severity of mucosal damage was assessed according to the following scoring system: 0 - no lesions or up to five puntiform lesions; 1 - more than five puntiform lesions; 2 - one to five small ulcers; 3 - more than five small ulcers or one large ulcer;4 - more than one large ulcer. 

**Table 1:** Ulcerogenic effect of diclofenac, lumiracoxib and lactam (1) in rats (n = 6,mean ± S.D.).

Compound	Number of ulcers	<1 mm	1-2 mm	>2 mm
diclofenac	74 ± 8.1	62 ± 6.3 (84%)	5.2 ± 1.8 (7%)	6.7 ± 2.4 (9%)
lumiracoxib	10 ± 1.2*	7.3 ± 2.1 (73%)	2.7 ± 1.1 (27%)	-
lactam **1**	0*	-	-	-

*significant difference compared to diclofenac group; P < 0.05 (Tukey’s test)

Diclofenac induced an average of 74 ulcerogenic lesions with around 6.7 (9%) lesions larger than2 mm. According to the scoring system, diclofenac was classified as class four. Lumiracoxib induced an average of 10 ulcerogenic lesions with around 2.7 (27%) lesions larger than 2 mm. This result allowed us to classify this drug as class two. The lactam **1** did not induce gastric ulceration and it was classified as class zero. Similar results were observed when diclofenac was converted to a lactam derivative [2]. These results demonstrated that the masking of carboxylic function of the lumiracoxib could decrease the gastro-ulcerogenicity. 

**Figure 2:**
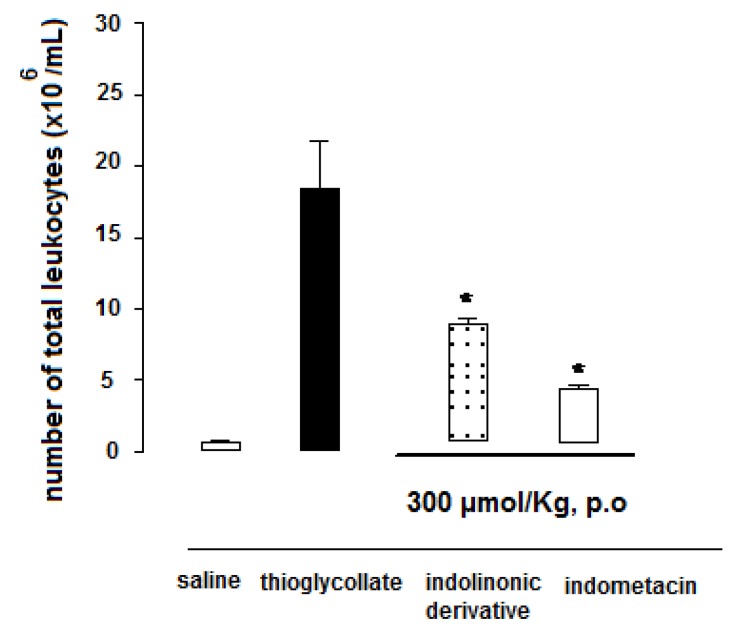
Effect of lactam derivative **1** and indomethacin on cell migration in peritonitis 3% thioglycollate-induced in mice. All compounds were administered p.o at a dose of 300 µmol/kg. Mice were killed at the time-point of 4 h after % thioglycollate-induced peritonitis. Total cell migration was counted using a Neubauer chamber. Data represent mean ± S.E.M. from at least six animals. * *P* < 0.01 (ANOVA followed by Dunnett’s test).

The thioglycollate-induced peritonitis in mice assay was performed in order to confirm the anti-inflammatory profile of lactam **1** suggested by the carrageenan-induced paw edema assay results. As depicted in Figure 2, the lactam derivative **1** was able to inhibit by 50.4% the cell migration using thioglycollate-induced peritonitis model. Indomethacin, used as drug control, presented 72% cell migration inhibition. In the same assay, lumiracoxib inhibited around 18% (data not shown).

#### 2.2.2. Analgesic activity

The lactam derivative’s ability to inhibit acetic acid-induced writhing in mice was studied in order to analyze its anti-nociceptive profile. The vehicle control exhibited 100% writhing, and the protection of the parental drug and its derivative was calculated on a percentage basis.

According to Table 2, lactam **1** offers analgesic protection 44.2% higher than that of lumiracoxib (38.8%). Indomethacin presented 73.1% of protection in the model used. These results suggest that 1-(2-chloro-6-fluorophenyl)-5-methylindolin-2-one (**1**) has anti-inflammatory and analgesic activity comparable with those of lumiracoxib. 

**Table 2:** Number of writhings and percentage of protection by indomethacin, lumiracoxib and lactam **1** at 100 μmol Kg^−1^, using the acetic acid induced writhing model in mice (n = 6, mean ± S.D.).

Treatment	Number of writhings (average)	% protection
control	60 ± 1.8	-
indomethacin	16.1 ± 1.1	73.1
lumiracoxib	36.7 ± 1.2*	38.8
lactam **1**	41.8 ± 0.7*	44.2

*statistical difference compared to control group. P < 0.05 (Tukey’s test)

#### 2.2.3. *In vitro* hydrolysis of 1-(2-chloro-6-fluorophenyl)-5-methylindolin-2-one (**1**) at pH 1.2 and 7.4

**Figure 3:**
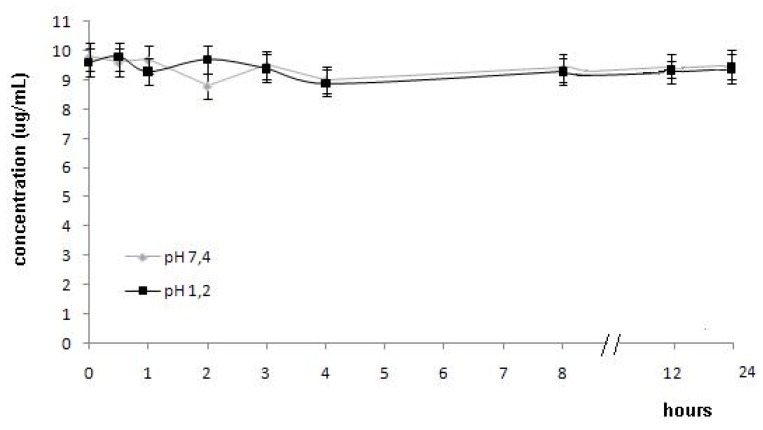
Hydrolysis of lactam derivatives (μg/mL) in buffer pH 1.2 and 7.4 (data are represented as mean ± S.E.M., *n* = 4, *P* < 0.05).

In order to observe the stability of lactam derivative **1** at the different pHs of gastrointestinal fluid and plasma, an HPLC (UV-Vis) method was developed. The hydrolysis study was carried out in aqueous buffer solutions (pH 1.2 and pH 7.4). The lactam derivative **1** was stable and did not hydrolyze at pH 1.2 and pH 7.4 during 24 h (Figure 3). These results indicated that stomach would not be exposed to carboxylic acid function of lumiracoxib (**2**). The absence of hydrolysis at pH 7.4 during 24 h indicated that lactam **1** could be absorbed in intact form. 

Previous reports demonstrated that this stability could be related to steric and electronic factors. Sterically, nucleophilic access to the lactam carbonyl group is blocked by fluorine and chlorine. This factor could explain the absence of lactam hydrolysis.

## 3. Experimental

### 3.1. General

Melting points were taken with an Electrothermal melting point apparatus (SMP3 Bibby Stuart Scientific) in open capillary tubes and they are presented as uncorrected values. Infrared spectra (KBr discs) were obtained on a Shimadzu FTIR-8300, and the frequencies were expressed in cm^−1^. ^1^H-NMR spectra were scanned on a Bruker DRX-400 (400 MHz) NMR spectrometer using DMSO-*d*_6 _ as solvent. Chemical shifts are expressed in ppm (parts per million) relative to tetramethylsilane. Elemental analysis (C, H and N) were obtained on a Perkin Elmer model 240C analyzer, and the data were within ± 0.4% of the theoretical values.

### 3.2. Materials

The lumiracoxib standard was donated by Novartis (São Paulo, Brazil). The diclofenac standard was donated by EMS-Sigma Pharma (Hortolândia, SP, Brazil). 1-Ethyl-3-[3-dimethylamino-propyl]carbodiimide hydrochloride (EDC, Sigma-Aldrich, St. Louis, MO, USA), potassium phosphate and trichloroacetic acid (TCA) were purchased from Labsynth (São Paulo, SP, Brazil). Acetonitrile was obtained from J.T. Baker (Phillipsburg, NJ, USA).

### 3.3. Animals

Male Wistar rats (200-250 g) and Swiss albino mice (25-30 g) were housed at a constant temperature (23 ± 1.8 ºC), humidity (55 ± 5%) and a light cycle (12/12 h) with food and water *ad libitum*. Experiments were conducted during the light phase. The study protocol was approved by The Research Ethics Committee of the School of Pharmaceutical Science, UNESP, Araraquara (Process 24/2010).

### 3.4. Synthesis of 1-(2-chloro-6-fluorophenyl)-5-methylindolin-2-one ***(1)***

Lumiracoxib (**2**, 315 mg, 1.07 mmol) and EDC (226 mg, 1.2 mmol) were placed in dichloromethane (20 mL) and the reaction mixture was stirred for 1 h at room temperature. Then, it was diluted with dichloromethane (50 mL) and washed with distilled water (3 × 20 mL). The organic phase was dried with sodium sulfate and the solvent removed by evaporation resulting in 251 mg (85% yield) of 1-(2-chloro-6-fluorophenyl)-5-methylindolin-2-one as a light yellow substance; m.p.: 130-132 °C. ^1^H-NMR: δ 2.35 (s, 3H), 3.87 (s, 2H), 6.83 (d, 1H, *J* = 7.67 Hz), 6.84 (d, 1H, *J* = 2.15 Hz), 6.85 (dd, 1H, *J* = 8.26 Hz and 2.1 Hz), 6.92 (ddd, 1H, *J* = 7.67 Hz), 7,02 (dd, 1H, *J* = 7.67 Hz and 1.5 Hz), 7.4 (d; *J* = 7.67 Hz, 1H) ppm; IR: 3,030 (C-H aromatic), 1,736 (C=O lactam), 1,458, 1,488 and 1,612 (C-C aromatic), 1,359 (C-N), 1,150 (C-F) and 783 (C-Cl) cm^−1^; MS-EI: 275 (*m/z*). Calculated for C_15_H_11_ClFNO: C, 65.35; H, 4.02; N, 5.08. Found: C, 65.50; H, 4.11; N, 5.30. 

### 3.5. Anti-Inflammatory Activity

The anti-inflammatory activity was evaluated using the carrageenan-induced rat paw edema method [7]. Wistar rats (150-200 g) were divided into three groups of six animals each. Group I served as a control group without using any drug, group II received lumiracoxib (**2**) at 300 μmol kg^−1^, and group III received lactam **1 **at 300 μmol kg^−1^ in a homogeneous suspension of sodium carboxymethyl-cellulose (0.5% w/v). The dose was molecularly equivalent to that of lumiracoxib. Each animal received orally 0.75-1.0 mL of the respective drugs. Thirty minutes after the administration of drugs, each rat received a subplantar injection of 0.1 mL of 1% carrageenan solution in its left hind paw. The measurement of the hind paw volume was carried out using a plethysmometer before each treatment (*V*o) and in each interval (*V*t) after the administration of drugs. All the results are expressed as mean ± S.E.M. Statistical analysis were performed with ANOVA followed by Tukey’s test.

### 3.6. Analgesic Activity

Analgesic activity was evaluated using 1% (v/v) acetic acid solution to induce writhing [8] in Swiss albino mice (25-30 g) of both sexes. The compounds were administered orally 1 h prior to acetic acid injections. The numbers of writhings were counted for 30 min duration in control and test compounds. Analgesic activity was measured as a decrease in writhings and compared with control. Mice were divided into four groups of six animals each. Group I served as a control group without any drug, group II received indomethacin (100 μmol kg^−1^), group III received lumiracoxib (100 μmol kg^−1^) and group IV received lactam derivative at 100 μmol kg^−1^. Each animal received 0.3-0.4 mL orally of the respective drugs, prepared as a homogeneous suspension of sodium carboxymethylcellulose (0.5% w/v). Acetic acid was administered intraperitoneally at a dose of 1 mL/100 g body weight. All the results are expressed as mean ± S.E.M. Statistical analysis were performed with ANOVA followed by Tukey’s test.

### 3.7. Ulcerogenicity

Gastrointestinal toxicity was determined using the method described by Cioli *et al.* [9]. The studies were carried out on healthy Wistar rats (150-200 g) at 300 μmol kg^−1^. The animals were divided into four groups of six animals each. Group I served as a control and received vehicle only, group II received pure diclofenac at 300 μmol kg^−1^, group III received lactam at 300 μmol kg^−1^and group IV received lumiracoxib at 300 μmol kg^−1^. The animals were fasted 8 h prior to receive the treatments, then they received free access to food and water, and they were sacrificed 17 h later. The rats’ gastric mucosa was examined using a 4 × binocular magnifier. The lesions were counted and divided into large (larger than 2 mm in diameter), small (1-2 mm) and puntiform (less than 1 mm). For each stomach, the severity of mucosal damage was assessed according to the following scoring system: 0 - no lesions or up to five puntiform lesions; 1 - more than five puntiform lesions; 2 - one to five small ulcers; 3 - more than five small ulcers or one large ulcer; 4 - more than one large ulcer. The mean score of each treated group minus the mean score of the control group was considered as the ‘severity index’ of gastric damage. Statistical analysis was performed with ANOVA followed by Tukey’s test.

### 3.8. Thioglycollate-Induced Peritonitis in Mice

The experiments were performed according to the method described by Savill *et al.* [10]. The animals were treated with lactam derivative **1** and indomethacin (300 μmol/Kg, p.o), and 1 mL 3% thioglycollate (Difco-BD Biosciences) i.p. was administered 1 h later. After 4 h, the animals were killed by cervical dislocation and the peritoneal cavity was washed with 1.5 mL cold PBS. The peritoneal exudates cells were retrieved and their volume was measured. The levels of significance between the experimental groups and the control were made using ANOVA in the tutorial Prisma®. The values were considered significant when *p < 0.05. The results were expressed as average ± S.E.M of the average.

### 3.9. In vitro Hydrolysis of 1-(2-chloro-6-fluorophenyl)-5-methylindolin-2-one (1) in buffer (pH 1.2 and 7.4)

#### 3.9.1. Analytical protocol

The concentrations of 1-(2-chloro-6-fluorophenyl)-5-methylindolin-2-one (**1**) and lumiracoxib (**2**) in buffer were measured by an HPLC method. The HPLC system used was a Shimadzu modelLC-10AD equipped with a model SPD-10A UV-Vis detector (Shimadzu). The compounds were separated on a Shimadzu Shim-pack CLCODS (M) reverse phase C_18_ column (5 μm particle size, 250 mm × 4.6 mm I.D) running an isocratic flow of 65% acetonitrile and 35% 25 mM sodium acetate/acetic acid aqueous buffer solution at pH 4.0, with a flow rate of 1.5 mL/min over 10 min and detection at 280 nm. The calibration curve was linear (r^2^ = 0.9999; n = 6) in the range 0.5-20 μg/mL. 

#### 3.9.2. *In vitro* hydrolysis of 1-(2-chloro-6-fluorophenyl)-5-methylindolin-2-one (1) in buffer (pH 1.2 and 7.4)

For buffer hydrolysis, an appropriate amount of pure 1-(2-chloro-6-fluorophenyl)-5-methylindolin-2-one (**1**) was weighed and diluted in sodium acetate (pH 1.2 and 7.4) to a concentration of 20 μg/mL. The samples solutions (20 μL) were injected into the HPLC system as follows: pH 1.2: zero; 0.5; 1.0; 2.0; 3.0; 4.0; 8.0; 12.0 and 24.0 h; pH 7.4: zero; 0.5; 1.0; 2.0; 3.0; 4.0; 8.0; 12.0 and 24.0 h. All samples were done in triplicate and the results are expressed by the average of the solutions concentrations.

#### 3.9.3. Statistical analysis

The data were expressed as mean ± SEM, and analyzed by one-way analysis of variance (ANOVA) followed by Tukey’s test for multiple comparisons among groups (Sigma-Stat software). The calibration curve and variation coefficients (CV %) were calculated using the Origin^®^ program.

## 4. Conclusions

The lactam derivative **1** presented anti-inflammatory and analgesic activity when compared to the lumiracoxib. The decrease of gastroulceration is the derivative’s main advantage and this effect could be related to the masking of the carboxyl group in the lactam function. Furthermore, the lactam derivative **1** is able to inhibit cell migration by 50.4% using a thioglycollate-induced peritonitis model, while lumiracoxib inhibited only 18%. The stability studies demonstrate that the lactam function is stable and does not hydrolyze at pHs 1.2 and 7.4. The discovery of new NSAID compounds with better safety and efficiency is important for the treatment of chronic inflammatory diseases. The next step is the pre-clinical pharmacokinetic and hepatotoxicity studies of the lactam derivative **1**.
